# Serial Functional and Genomic Analyses Illuminate Clonal Evolution in Metastatic NSCLC with 12-Year Survival

**DOI:** 10.3390/curroncol32110646

**Published:** 2025-11-19

**Authors:** Vikrant S. Bakaya, Sabina A. Schneider, Tracy Nguyen, Derrick C. Phu, Lucas A. Alvarez, Steven S. Evans, Paula J. Bernard, Federico R. Francisco, Adam J. Nagourney, Luisa Torres, John Henry, Paulo D’Amora, Robert A. Nagourney

**Affiliations:** 1 Nagourney Cancer Institute, 750 East 29th Street, Long Beach, CA 90806, USA; vbakaya@nagourneyci.com (V.S.B.); saschneider5@wisc.edu (S.A.S.); tnguy294@jh.edu (T.N.); derrickphu@g.ucla.edu (D.C.P.); lucasa@bu.edu (L.A.A.); sevans@nagourneyci.com (S.S.E.); pbernard@nagourneyci.com (P.J.B.); ffrancisco@nagourneyci.com (F.R.F.); anagourney@nagourneyci.com (A.J.N.); ltorres@nagourneyci.com (L.T.); jhenry@nagourneyci.com (J.H.J.); pdamora@nagourneyci.com (P.D.); 2 Metabolomycs, Inc., 750 E. 29th Street, Long Beach, CA 90806, USA; 3 Department of Obstetrics and Gynecology, University of California Irvine (UC Irvine), 101 The City Dr S, Orange, CA 92868, USA

**Keywords:** non-small cell lung cancer, clonal evolution, functional profiling, *BRAF* mutation, *EGFR* mutation, long-term survival

## Abstract

**Background:** Non-small cell lung cancer (NSCLC) is the most common form of lung cancer and a leading cause of cancer-related death. Despite therapeutic advances, long-term survival in stage IV disease is uncommon. Tumor analyses that combine genomic and functional platforms may provide the opportunity to monitor clonal dynamics and guide therapy selection. **Case Presentation:** We report a 67-year-old woman with metastatic poorly differentiated lung adenocarcinoma, who achieved four durable remissions and survived nearly 12 years. Serial studies using ex vivo analysis of programmed cell death (EVA/PCD) functional-profiling-guided therapeutic choices were correlated with next-generation sequencing (NGS). Molecular events included the emergence of a *BRAF* V600E mutation responsive to dabrafenib plus trametinib and the acquisition of an *EGFR* exon 19 deletion responsive to Osimertinib. EVA/PCD identified activity for targeted agents and revealed synergy for vinorelbine plus Osimertinib not predicted by genomic profiling, which provided additional response. **Discussion:** This case highlights clonal evolution in NSCLC and illustrates how serial tissue analyses correlating phenotypic and genomic events can offer therapeutic interventions to provide long-term survival. **Conclusions:** The integration of functional and genomic profiling may improve personalized treatment in NSCLC by interrogating tumor heterogeneity and clonal evolution to inform rational therapeutic selection.

## Simple Summary

The majority of non-small cell lung cancer patients present with advanced disease, whereby survival is less than 2 years. We report a woman with metastatic non-small cell lung cancer who survived 12 years, benefiting from serial tissue analyses that applied genomic and functional platforms to select therapies. Each recurrence manifested distinct biologic features that responded to drugs selected by genotypic and phenotypic analyses. Following laboratory-directed chemotherapy and subsequent immunotherapy, the patient was found positive for a *BRAF* V600E mutation and responded to Dabrafenib plus Trametinib. Subsequent progression revealed an *EGFR* del19 mutation, not present in initial genomic analysis, as the previously identified *BRAF* mutation disappeared. Serial interrogations identified clonal expansions with distinct phenotypes. Each intervention selected highly adapted resistant subpopulations that revealed new therapeutic vulnerabilities. The case represents the application of serial tissue analyses to identify therapies, offering hope for more effective personalized cancer strategies in the future.

## 1. Introduction

Lung cancer is the third most common malignancy in the United States, with an estimated 226,650 new diagnoses and 124,730 deaths projected in 2025 [1]. Non-small cell lung cancer (NSCLC) accounts for over 80% of cases, with adenocarcinoma being the predominant subtype [2]. While lung cancer has historically been associated with tobacco use [3], the incidence in non-smokers has been steadily rising now, accounting for 10–25% of all new cases [4]. An examination of the mutagenic events driving this increase identified air pollution as a principal cause [5]. As somatic mutations are increasingly recognized as drivers of oncogenesis [6], lung cancer has been subclassified based upon specific mutational drivers [7]. Today, molecular testing for driver mutations and gene rearrangements—such as *EGFR*, *ALK*, and *KRAS*—has become the standard, enabling the use of targeted therapies that have transformed outcomes in select subgroups.

Tumorigenesis in NSCLC is increasingly understood as an evolutionary process shaped by clonal selection and environmental pressures [8, 9, 10]. The TRACERx trials have demonstrated how circulating tumor DNA (ctDNA) can be used to map phylogenetic evolution and track resistance mechanisms [11].

Prior efforts to measure drug response using human tumor primary cultures focused primarily upon drug-induced tumor cell growth inhibition using clonogenic [12] or DNA synthesis endpoints [13]. With the recognition of apoptosis as a fundamental driver of tumorigenesis [14], newer laboratory models have employed drug-induced cell death to predict drug response. Among the cell death platforms, tumor organoids [15], explant cultures [16], and ex vivo analysis of programmed cell death (EVA/PCD) [17, 18, 19] have been successfully employed. Together with genomic analyses, primary culture models can provide complementary insights into tumor biology and therapeutic vulnerabilities.

Here, we describe the case of a patient with T2N3M1 metastatic NSCLC, whose nearly 12-year survival was achieved through serial integration of functional and genomic profiling.

## 2. Case Presentation

A 67-year-old woman presented to medical attention in August 2013 with a 2-month history of cough. The patient was nonsmoker but had a history of secondhand smoke exposure from her husband. Past medical history included hypercholesterolemia, hypertension, hypothyroidism, and hysterectomy for dysfunctional bleeding. The patient denied chest pain, weight loss, or hemoptysis.

A CT scan conducted on 26 August 2013 identified multiple pulmonary nodules, mediastinal and hilar adenopathy, and a 3.3 cm cavitary lesion in the left upper lobe. A CT-guided biopsy of the dominant left lung mass conducted on 30 August 2013 confirmed moderately to poorly differentiated adenocarcinoma found to be CK7- and TTF1-positive. Tumor was negative for *EGFR* mutation and *ALK* gene rearrangement.

Treatment Cycle I: On 13 September 2013, confirmatory biopsy provided tissue for the ex vivo analysis of programmed cell death (EVA/PCD) that utilized tumor explants isolated from the surgical specimen. Results identified the optimal regimen to be the combination of Carboplatin plus Paclitaxel plus Irinotecan based upon the triplet’s activity and synergy compared with each single agent using isobologram synergy analysis [20, 21]. The patient received treatment every 3 weeks from 20 September 2013 through 20 December 2013, completing six cycles. A PET/CT on 27 December 2013 confirmed partial response and the patient was then maintained with Pemetrexed every 3 weeks for four cycles, in keeping with established maintenance schedules employed in NSCLC patients following response to platinum-based chemotherapy [22].

Treatment Cycle II: Increasing cough and a rising CEA led to repeat CT scan in September of 2014, confirming growth in the lung nodules and mediastinal nodes and a new adrenal metastasis. The patient was referred to UCLA for enrollment in the Keynote 010 Pembrolizumab trial [23]. Immunohistochemistry performed upon the original biopsy was reported by UCLA as PDL-1 positive but records from the original analysis are no longer available for review. The patient achieved disease control through August 2016, when PET/CT of 15 August 2016 confirmed progression in the left upper lobe and new left supraclavicular adenopathy.

Treatment Cycle III: Biopsy of the left clavicular node on 26 August 2016 was submitted for EVA/PCD that identified activity for the *BRAF* inhibitor Vemurafenib and the *MEK* inhibitor Selumetinib. Foundation One next generation sequence (NGS) was positive for *BRAF* V600E and negative for *RET*, *ALK*, *EGFR*, *KRAS*, *ERBB2,* and *MET*. Based upon the recently reported success of the closely related doublet of Dabrafenib plus Trametinib in relapsed *BRAF* (+) NSCLC [24] treatment with oral Dabrafenib plus Trametinib was instituted on 22 September 2016, providing durable remission through January 2020. Her management was complicated by the development of paraneoplastic autonomic failure (PAF) successfully managed with a combination of corticosteroids and intravenous immunoglobulin (IVIG).

During a hospitalization for diverticular abscess with colo-vesical fistula, a CT scan conducted on 2 January 2020 revealed growth of the left upper lobe nodule which led to biopsy on 3 January 2020. Tissue submitted for EVA/PCD revealed activity for Gefitinib, the *EGFR* Tyrosine Kinase Inhibitor (*TKI*).

Foundation One NGS analysis conducted on the tissue confirmed a previously undetected *EGFR* p.Thr751_Thr759delinsAsn mutation, while the previously identified and treated *BRAF* V600E mutation became undetectable.

Treatment Cycle IV: Based upon the results of the FLAURA trial [25], therapy with oral Osimertinib at 80 mg per day begun on 26 February 2020 and that provided response through May of 2022 when CT scan on 20 May 2022 confirmed progression in the left upper lobe of the lung. A biopsy was submitted for Foundation One NGS confirmed the *EGFR* del19 and provided tissue for EVA/PCD analysis.

Prior observations that identified synergy for Vinorelbine combined with the *EGFR* tyrosine kinase inhibitor Gefitinib [26], and reported activity for this doublet in NSCLC [27] and the successful application of this combination in advanced malignancies [28] led to the ex vivo evaluation of Osimertinib plus Vinorelbine that revealed activity and synergy for the doublet.

Treatment Cycle V: Oral Osimertinib was continued at 80 mg per day and combined with Vinorelbine at 20 mg/m^2^ IV on day 1 and 8 q on day 21 (off-label), which provided a response. This was followed by consolidation with five doses of SBRT to the left upper lobe nodule completed on 11 March 2023. A PET/CT conducted on 23 February 2024 revealed subtle abnormalities in vertebral body of T5 and the sacrum without significant metabolic activity, consistent with controlled metastatic disease.

New neurological symptoms led to an MRI of the brain on 20 March 2024. When compared with prior MRI of 23 October 2023, the MRI identified two new left frontal brain metastases. The patient received cyber knife radiation with disease control but suffered radiation necrosis resulting in aphasia and right-sided weakness.

Repeat PET/CT on 31 May 2024 revealed “no focal area of hypermetabolic activity suspicious for residual or metastatic hypermetabolic neoplastic pathology. Findings of the current study are most consistent with complete response to treatment administered for metastatic lung carcinoma.” and the patient continued the Osimertinib-based regimen.

**Figure 1:**
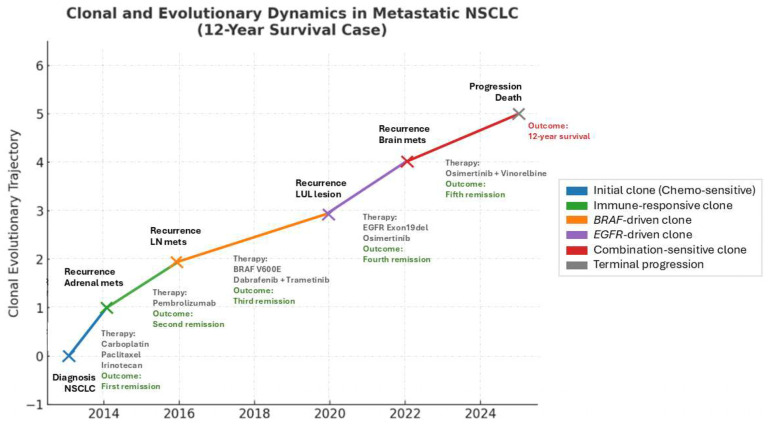
Clonal and evolutionary trajectory of NSCLC case, with treatments and outcomes mapped over time. Each colored branch represents the rise in a dominant clone under selective pressure. Therapies and outcomes are annotated along the trajectory. NSCLC (non-small cell lung cancer); LN mets (lymph node metastasis); LUL lesion (left upper lobe lung lesion).

On 24 October 2024, due to gait instability, the patient suffered a traumatic fracture of the right hip requiring internal fixation and revision on 18 February 2025. The patient’s condition continued to deteriorate and she expired on 26 February 2025, nearly 12 years after initial diagnosis. The clonal and therapeutic dynamics described above are summarized in Figure 1, which illustrates the evolutionary trajectory of the tumor alongside treatments and clinical outcomes.

## 3. Discussion

This case illustrates how the integration of phenotypic and genomic analyses can interrogate clonal evolution in metastatic NSCLC and extend survival. Functional profiling identified activity for targeted agents including *BRAF* and *EGFR* inhibitors that was then correlated with genomic analyses using NGS. Functional profiling also revealed synergistic strategies including carboplatin/paclitaxel/irinotecan and Vinorelbine/Osimertinib that were not readily evaluable by genomic analyses.

The patient’s clinical course reflects successive clonal expansions following each therapy, whereby dominant clones were suppressed and replaced by resistant sub-clones. This aligns with Darwinian selection [29] but the observed shifts in tumor biology are also consistent with Gould’s model of punctuated equilibrium [30], whereby catastrophic environmental alterations mediated by chromothripsis and chromoplexy related to drug exposure cause abrupt changes in tumor phenotype [8]. It has been suggested that individual tumors may follow more than one evolutionary pathway depending upon the type of mutational event, i.e., point mutations versus copy number alterations [31]. Furthermore, the type of treatment may influence the type of mutation observed as cisplatin induces single and dinucleotide substitutions [32], while APOBEC may underlie evolutionary response to other stressors [33]. The recognition that intra-tumoral heterogeneity is present at the time of initial diagnosis [34], coupled with epigenetic plasticity [27, 35, 36, 37], may have contributed to the tumor’s adaptive trajectory.

## 4. Conclusions

The integration of serial functional and genomic profiling can enhance therapeutic decision-making in metastatic NSCLC and reveal effective strategies for treatment. This patient’s 12-year survival underscores the potential of phenotypic analyses combined with precision genomics in an era of personalized oncology.

## Data Availability

Data utilized in the preparation of this manuscript is reported in the manuscript.
